# A Brief Review on the Biology and Effects of Cellular and Circulating microRNAs on Cardiac Remodeling after Infarction

**DOI:** 10.3390/ijms22094995

**Published:** 2021-05-08

**Authors:** Mihir Parikh, Grant N. Pierce

**Affiliations:** Institute of Cardiovascular Sciences, St. Boniface Hospital and the Department of Physiology and Pathophysiology, College of Medicine, Rady Faculty of Health Sciences, University of Manitoba, Winnipeg, MB R2H 2A6, Canada; mihir.parikh@mssm.edu

**Keywords:** circulating miRNA, exosomes, miR-1, miR-133, miR-135, miR-29, cardiac remodeling

## Abstract

Despite advances in diagnostic, prognostic, and treatment modalities, myocardial infarction (MI) remains a leading cause of morbidity and mortality. Impaired cellular signaling after an MI causes maladaptive changes resulting in cardiac remodeling. MicroRNAs (miRNAs/miR) along with other molecular components have been investigated for their involvement in cellular signaling in the pathogenesis of various cardiac conditions like MI. miRNAs are small non-coding RNAs that negatively regulate gene expression. They bind to complementary mRNAs and regulate the rate of protein synthesis by altering the stability of their targeted mRNAs. A single miRNA can modulate several cellular signaling pathways by targeting hundreds of mRNAs. This review focuses on the biogenesis and beneficial effects of cellular and circulating (exosomal) miRNAs on cardiac remodeling after an MI. Particularly, miR-1, -133, 135, and -29 that play an essential role in cardiac remodeling after an MI are described in detail. The limitations that will need to be addressed in the future for the further development of miRNA-based therapeutics for cardiovascular conditions will also be discussed.

## 1. Introduction

### 1.1. microRNAs and Cardiovascular Disease

Several microRNAs (miRNAs/miR) have been involved in cardiac pathological processes, such as acute myocardial infarction (MI), cardiac arrhythmias, and hypertrophy [1]. The role of miRNAs is increasingly being recognized as diagnostic, prognostic markers, or therapeutic targets for cardiovascular diseases. A study by Da Costa Martins et al. [2]. reported that genetic deletion of a nuclease enzyme involved in miRNA biogenesis resulted in maladaptive cardiac remodeling and heart failure. This underlines the importance of miRNAs for cardiac function. MiRNAs can either promote or inhibit the apoptosis of cardiomyocytes, modulate angiogenesis, alter cardiac regeneration, and/or re-program cardiac fibroblasts into cardiomyocytes [3]. These effects clearly support a relationship between altered miRNA expression and ischemic heart disease.

### 1.2. Biology of microRNAs

**Figure 1:**
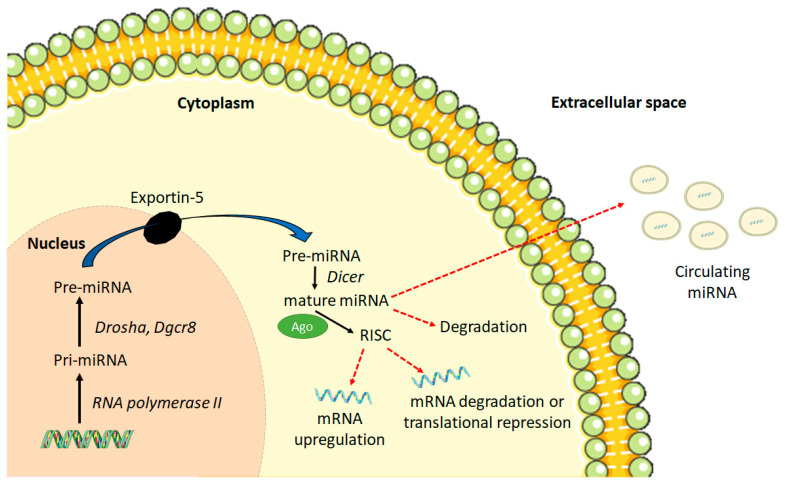
Schematic of miRNA biogenesis and function. AGO, Argonaute protein; RISC, RNA-induced silencing complex.

miRNAs are endogenous, noncoding, single-stranded RNAs 22–26 nucleotides in length that inhibit messenger RNA (mRNA) expression through the Watson–Crick base pairing between sequences located in the three prime untranslated regions (3′ UTR) of mRNA and miRNA [1,3]. MiRNAs are transcribed by RNA polymerase II as pri-miRNAs, which are ~2 kb in length. In the nucleus, pri-miRNAs are cleaved by the RNase III-type enzyme *Drosha*, *Dgcr8*, and other factors to produce a short stem-loop structure of ~70 nucleotides called pre-miRNAs [4] (Figure 1). They are then exported out of the nucleus through exportin-5 into the cytoplasm where they undergo further processing by the ribonuclease *Dicer*, producing a double-stranded RNA molecule [3].

One strand of the miRNA duplex is selected as mature miRNA and the other one, known as the “passenger strand”, is eliminated and degraded [3,5]. This selection process is determined by the strength of base pairing between the two strands of miRNA duplex [5]. These then associate with the Argonaute protein to form the RNA-induced silencing complex (RISC) [3]. miRNAs in RISC can bind to specific mRNAs by identifying miRNA recognition elements (seeds) located in the 3’ UTR of target mRNAs. The nucleotides at the 2-8 position at the 5’-end of the miRNAs must be complementary to the ‘seed’ region of the target mRNAs for miRNAs to exert their functional effects of translational depression or direct degradation of the target mRNAs [1,3]. A greater degree of complementarity between miRNAs and target mRNAs is more likely to result in target mRNA degradation [6]. Increasing evidence has also suggested that in addition to the target mRNA degradation or translational repression, miRNAs can stabilize and upregulate their target mRNA expression [7]. This miRNA-mediated positive gene regulation is a cell-type and condition-specific process. It could occur through the direct action of miRNA/miRNA-associated ribonucleoproteins (miRNPs) or through indirect mechanisms whereby the repressive effects of miRNA/miRNPs are abrogated [7,8].

The miRNAs can either bind to target mRNA and undergo degradation or can be released from cells through the following export mechanisms [9,10]. The pre-miRNA can be incorporated into multivesicular bodies that fuse with the plasma membrane and are then released into the extracellular space and circulation in the form of exosomes. The cytoplasmic miRNAs are also secreted from the cells as microvesicles through membrane blebbing or shedding. In addition, they can be released in the form of apoptotic bodies by some cell types such as endothelial cells. The circulating miRNAs can thus be found either in a “free” form complexed with proteins or as membrane-bound bodies [9,10]. Circulating miRNAs are derived from the heart, liver, lung, kidney, and blood cells [6]. As the circulating miRNAs reside either in the exosomes, microvesicles, or apoptotic bodies, they are protected against degradation by the endogenous RNase activity [6]. This stability of circulating miRNAs is being exploited to use them as diagnostic and prognostic biomarkers for diseases. 

The human genome is estimated to encode approximately 1000 miRNAs that can target hundreds of distinct mRNAs [6]. Based on the genomic locations of miRNA genes, miRNAs are classified into the following four types: (a) the intergenic miRNAs, which are transcribed from their own genes; (b) the intronic miRNAs, which are transcribed from the introns of their host protein-coding genes; (c) the exonic miRNAs, which are transcribed from the exons of their host protein-coding genes; and (d) the untranslated region miRNAs, which are transcribed from the 3’ or 5’ UTR of the protein-coding genes [11]. Epigenetic mechanisms can also regulate the transcription of miRNAs [12]. 

### 1.3. MI and microRNAs

Cardiac injury during a myocardial infarction (MI) is caused by ischemic and hypoxic conditions. An MI can cause cardiac cell death; impaired cardiac signaling, which can lead to infarct expansion; amplified oxidative stress; and the remodeling of surviving cardiomyocytes. Cardiac remodeling starts as an adaptive mechanism to maintain heart function. However, this process becomes maladaptive in pathological conditions, and, consequently, cardiac fibrosis, dilated cardiomyopathy, and heart failure develop. Cardiac remodeling is a complex pathological condition where many molecular components are involved. At present, many investigations are focusing on dissecting the molecular mechanisms involved in post-infarction remodeling and heart failure. Endogenous miRNAs have important roles in various cardiovascular pathologies [5,13]. Altered levels of miRNA have been reported in ischemic/reperfused hearts in animal and human studies. He et al. [14] observed an upregulation of 10 miRNAs and downregulation of 6 miRNAs in Sprague–Dawley rat hearts subjected to 1 h ischemia followed by 3 h reperfusion. In a total coronary occlusion rat model of myocardial infarction, Dong et al. [15] reported differential expression of 38 miRNAs in the infarcted zone and 33 miRNAs in the border zone compared with the non-infarcted zone. Bostjancic et al. [16] examined infarcted heart tissue from the MI patients and found 77 dysregulated miRNAs, of which 47 miRNAs changed within 1-week post-MI and 30 miRNAs were altered 4 weeks post-MI. Impairment in the function of ion channels, transporter proteins, intracellular calcium handling proteins, and other relevant proteins during an MI can create substrates that can predispose the heart to abnormalities in electrical conduction and arrhythmias. Recent studies have highlighted the importance of miRNAs in the regulation of cardiac rhythm [3]. Some of the miRNAs that play important roles in the cardiac remodeling and arrhythmias that occur following an MI are discussed in the following section and Table 1. 

#### 1.3.1. microRNA-1 

miR-1 is one of the most abundant miRNAs expressed in the myocardium [31]. Its overexpression is observed in coronary artery disease patients [32]. The miR-1 family consists of an miR-1 subfamily containing miR-1-1 and miR-1-2 transcripts. However, these two subfamily members are located on two separate chromosomal regions, 20q13.33 and 18q11.2, respectively [31]. 

An approximately 2.6-fold increase in miR-1 expression was accompanied by arrhythmias in a rat MI model of permanent coronary artery ligation [3]. Increased expression of miR-1 results in a widening of the QRS complex, indicating slowed cardiac conduction, which explains the role of miR-1 as an arrhythmogenic agent in ischemic hearts. miR-1 post-transcriptionally inhibits the potassium voltage-gated channel subfamily J member 2 (*KCNJ2*), which codes for the Kir2.1 protein. Kir2.1 is the subunit of the K+ channel, which carries the inward rectifier potassium current [3]. A reduction in Kir2.1 prolongs the action potential duration and QT interval, resulting in higher incidences of ventricular tachyarrhythmia [31]. miR-1 represses the gap junction protein alpha 1 *(GJA1),* which encodes the connexin-43 gap junction channel protein, thus slowing down the cardiac conduction in the setting of an MI [32]. Other targets of miR-1 include the notch ligand delta, the Rho GTPase *Cdc42*, Iroquois homeobox domain 5 (*Irx5*), and Shal-related family member 2 (*KCND2*) [33]. 

These cellular mechanisms could be responsible for the arrhythmogenic potential of miR-1. Consistent with this hypothesis, the introduction of miR-1 worsened arrhythmias, while suppression through antisense inhibitors reduced arrhythmias in rats undergoing an MI [33]. Moreover, propranolol, a β-blocker used as an antiarrhythmic agent, can downregulate miR-1 expression and thus improve cardiac conduction [34]. Interestingly, the expression levels of miR-1 in the left atrial tissue from patients with atrial fibrillation were reduced. This differential regulation of miR-1 in the left atrial and ventricular tissues suggests that the miR-1 levels in the myocardium should be maintained within a proper range [3]. However, further studies are warranted to understand the effect of miR-1 expression in different tissues and conditions. The induction of the miR-1 expression in the rat model of MI lowered the expression of insulin-like growth factor-1 [35] and other anti-apoptotic genes, such as B-cell lymphoma-2 (Bcl-2) and heat shock protein (HSP) -60 and -70 [13]. This could be responsible for the activation of the pro-apoptotic pathways in cardiomyocytes, which may ultimately impact left ventricular remodeling. 

#### 1.3.2. microRNA-133

miR-133 is dominantly expressed in cardiomyocytes and cardiac fibroblasts (19). Its targeted deletion, overexpression, and antisense-mediated knockdown have demonstrated its essential roles and targets in cardiac remodeling [36]. The miR-133 family has two isoforms, *-*133a and -133b. Both isoforms have a similar sequence except for the 3’-terminal base, where guanidine is found in miR-133a and adenosine in miR-133b [13]. The miR-133a isoform has two further subvariants, -133a-1 and -133a-2. Depending upon the species, these miRNAs are located on different chromosomes. For example, in the murine genome, miR-133a-1 is arranged with miR-1-2 on chromosome 18, mir-133a-2 is clustered with miR-1-1 on chromosome 2, and miR-133b is located with miR 206 on chromosome 1. However, in the human genome, miR-133a-1, miR-133a-2, and miR-133b are located on chromosomes 18, 20, and 6, respectively [37]. 

miR-133 and miR-1 are transcribed together; however, whereas miR-1 is pro-apoptotic, miR-133 has an anti-apoptotic role [38]. In both in vitro and in vivo models of ischemia and reperfusion injury, overexpression of exogenous miR-133a reduced the apoptosis of cardiomyocytes [39]. The β-blocker carvedilol protected cardiomyocytes against apoptosis by upregulating miR-133 expression [40]. miR-133 exerts its action by inhibiting pro-apoptotic genes, such as death-associated protein kinase 2 (DAPK2), apoptotic protease activating factor 1 (APAF1), caspase-9, Bcl-2 like 11, and Bcl-2-modifying factor (BMF) [13]. 

A downregulation of miR-133a levels in a rat model of MI [13] and in cardiac tissue from MI patients has been observed [41]. Conversely, circulating levels of miR-133a in blood have been upregulated in acute MI patients, which could suggest cardiac damage [42]. The levels of circulating miR-133a are sensitive to cardiac injury and are elevated before changes in cardiac troponin T (cTnT) or creatine phosphokinase (CPK) can be observed [43]. This implies that miR-133a could provide strong diagnostic and prognostic information compared to the traditional markers [44]. In a rat model of MI, the overexpression of miR-133 increased left ventricular ejection fraction and fractional shortening [45]. Overexpression of miR-133a has improved cardiac function by decreasing myocardial fibrosis in the streptozotocin-induced diabetic cardiomyopathy in mice [46] and heart failure in rats [47]. miR-133a exerted its anti-fibrotic effect by inhibiting the expression of profibrotic genes such as connective tissue growth factor (CTGF) and collagen 1A1 [48] as well as suppression of the upregulated Akt-dependent signaling pathways in heart failure [47]. Inflammation plays a critical role in the adverse left ventricular remodeling after an MI [49]. Overexpression of miR-133a reduced the inflammatory cell infiltration in the myocardium after an MI [50]. miR-133a can also be cardiogenic by reprogramming human and murine fibroblasts via suppressing Snai1, a key modulator of epithelial to mesenchymal transition [51]. It can also induce transdifferentiation of cardiac fibroblasts by inhibiting the transforming growth factor-β (TGF-β) signaling cascade [50]. miR-133 has also been studied for its correlation with the incidences of ventricular fibrillation (VF). The downregulation of miR-133a/b was associated with the development of VF in MI patients by increasing the expression of hyperpolarization-activated cyclic nucleotide-gated ion channel 2 (HCN2), which determines cardiac automaticity. This indicates that increased expression of miR-133a/b could be used therapeutically for reducing the incidence of arrhythmias in MI patients [52]. 

In summary, because of its anti-fibrotic, anti-apoptotic, and regenerative potential, miR-133a could be further explored therapeutically for its cardioprotective and regenerative effects after cardiac injury. 

#### 1.3.3. microRNA-29

The miR-29 family consists of miR-29a, miR-29b-1, miR-29b-2, and miR-29c [53]. Despite being transcribed from different genetic loci, both miR-29b-1 and miR-29b-2 have identical mature sequences [54]. Thus, both are collectively termed as miR-29b. In the human genome, miR-29a and miR-29b-1 are located on chromosome 7, whereas miR-29b-2 and miR-29c are on chromosome 1 [54]. A study has demonstrated that miR-29b is the dominant member of the miR-29 family as its myocardial expression is 4- and 8-fold higher than that of miR-29a and miR-29c, respectively [55]. miR-29 is an essential regulator of extracellular matrix proteins and pathways that are related to fibrosis [53]. The miR-29 family members target 16 genes that code for extracellular matrix proteins, such as collagen isoforms, fibrillin 1, elastin, matrix metalloproteinase 2, laminin γ1, and integrin β1 [56,57]. 

miR-29 is highly expressed in fibroblasts, and its inhibition can induce collagen expression in both cell culture and mice after an MI [2]. This demonstrates the profibrotic effect of lowering miR-29 expression. A study by Zhang et al. [58] demonstrated the antifibrotic effect of miR-29b in angiotensin II-induced cardiac fibrosis in mice. Consistent with this, carvedilol produced an antifibrotic effect in a murine model of MI by upregulating miR-29b [59]. However, Sassi et al. [60] demonstrated that miR-29 promotes rather than reduces cardiac fibrosis. However, these contrasting data could be explained by the use of a mouse model for left ventricular pressure overload-induced by transverse aortic constriction (TAC) by Sassi et al. [60] unlike other studies [53,61] that used a coronary artery ligation-induced animal model of MI. There are differences in the type of cardiac fibrosis produced in these experimental models [62]. The cardiac fibrosis in TAC is mainly characterized as reactive interstitial fibrosis, which occurs as an adaptive mechanism to preserve cardiac structure and function. In contrast, in the ischemic injury model, replacement fibrosis occurs to fill up the void of viable cardiomyocytes to prevent cardiac rupture. In addition, other studies have shown the beneficial effects of the upregulation of a specific variant of miR, -29b, while Sassi et al. [60] assessed the global deletion of miRNA regardless of variant subtypes. Therefore, these experimental differences could be responsible for the contrasting results in these studies.

An upregulation of miR-29b improved cardiac function in doxorubicin-induced cardiotoxicity in rats [55]. The improved cardiac function by miR-29b has been attributed to its inhibitory effect on the apoptosis of cardiomyocytes. Consistent with this, overexpression of miR-29b promoted apoptosis of cardiomyocytes in vitro, and downregulation of miR-29b by an miR-29b inhibitor suppressed apoptosis of cardiomyocytes in vitro [55]. The exact mechanism behind this anti-apoptotic effect of miR-29b has been identified. It targeted the Bax 3’ UTR to reduce Bax expression, which is a pro-apoptotic protein. It also induced the expression of Bcl-2, an anti-apoptotic protein. Furthermore, it inhibited mitochondrial membrane depolarization, cytochrome C release, and activation of caspase-3 [55]. miR-29b has also been shown to suppress tumor necrosis factor-related receptor 5 (TRAF5) to exert its anti-apoptotic effect in hypoxia-induced cardiomyocyte injury [63]. miR-29b may also improve cardiac function through the activation of Akt in endothelial cells that promotes endothelial cell-mediated angiogenesis [61]. The induction of angiogenesis in MI is known to improve cardiac function and decrease the risk of cardiac rupture [64].

Increasing evidence now suggests that the downregulation of miR-29 contributes to the development of cardiac fibrosis. In this setting, miR-29 mimics that could elevate the expression levels of miR-29 or, alternatively, pharmacological inhibitors that could prevent the downregulation of miR-29 might represent effective therapeutic strategies [53].

#### 1.3.4. microRNA-135

miR-135 is comprised of two variants, miR-135a (miR-135a-1 and miR-135a-2), and miR-135b [65]. miR-135 is less widely studied in the field of cardiovascular physiology and pathophysiology. miR-135a is encoded by two genes located on chromosome 3 and 12, whereas miR-135b is found on chromosome 1 [66]. miR-135b is expressed 10-fold less in the midbrain raphe nuclei than miR-135a [65]. Chu et al. [67] reported a reduction in cardiac hypertrophy by the overexpression of miR-135b in both angiotensin II and TAC-induced models. *CACNA1C*, a gene that codes for L-type calcium channels, was the main target of miR-135b [67]. It was also predicted to target phosphodiesterase 1A (PDE1A), and the cardiac-specific sodium–calcium exchanger (NCX1) [67]. miR-135b improved cardiac function in a murine MI model and protected cardiomyocytes in vitro by downregulating the expression of caspase-1, NOD-like receptor-containing pyrin 3 (NLRP3), and interleukin-1β (IL-1β) [68].

miR-135a expression levels are reduced in myocardial ischemia–reperfusion injury in diabetic mice [69]. Upregulation of miR-135a was achieved by using mimics in this model. This reduced the myocardial infarct size and apoptosis by decreasing the expression of the thioredoxin-interacting protein (TXNIP) [69]. TXNIP (also called the thioredoxin-binding protein-2 or vitamin D3 upregulated protein-1) regulates the cellular redox state and is associated with apoptosis [70]. Wang et al. [71] reported that upregulation of miR-135a decreased myocardial infarct size and apoptosis in an ischemia–reperfusion injury model. Protein tyrosine phosphatase 1B (PTP1B), which plays an important role in several cellular processes, such as differentiation, proliferation, migration, and apoptosis, is a direct target of miR-135a [71]. A negative association between PTP1B and miR-135a was observed [71]. Wu et al. [72] demonstrated that the antifibrotic effect of miR-135a is mediated through the transient receptor potential melastatin 7 (TRPM7) channel, which plays a crucial role in cellular proliferation and differentiation.

Duoung et al. [73] reported the downregulation of miR-135a in the left ventricle of mice with a complete atrioventricular block induced by His-bundle ablation. The sodium–calcium exchanger type 1 (NCX1) has been identified as the main target of miR-135a [73]. Increased expression and activity of the NCX1 that bi-directionally exchanges 3 Na^+^ for 1 Ca^++^ are adaptive mechanisms during the cardiac remodeling process to maintain cardiac function. However, this alteration can become pro-arrhythmic and produce early- and delayed-after depolarizations as a consequence [73]. To support this, overexpression of miR-135a reduced the spontaneous beating frequency of rat neonatal cardiomyocytes by 63% [73]. miR-135a should be explored further to exploit its anti-arrhythmic potential for modulating cardiac automaticity, spontaneous calcium release, and calcium efflux.

**Figure 2:**
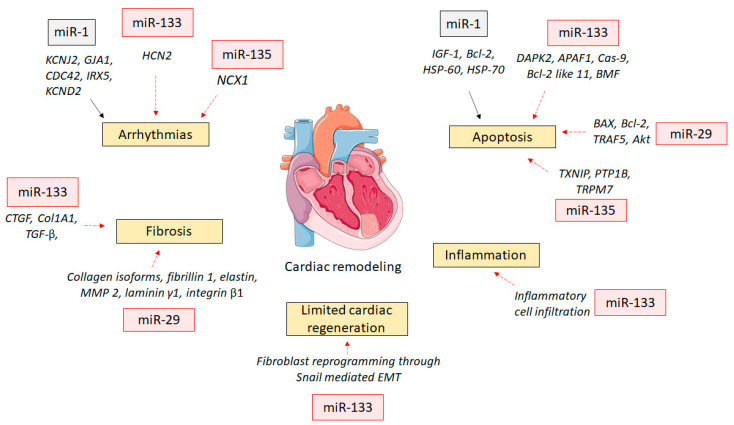
Potential role of miRNAs in positively impacting cardiac remodeling after a myocardial infarction. Akt, protein kinase B; APAF1, apoptotic protease-activating factor 1; BAX, Bcl-2-associated X-protein; Bcl-2, B-cell lymphoma-2; BMF, Bcl-2-modifying factor; Cas-9, caspase-9; CDC42, cell division control protein 42 homolog; COL1A1, collagen 1A1; CTGF, connective tissue growth factor; DAPK2, death-associated protein kinase 2; EMT, endothelial mesenchymal transition; GJA1, gap junction protein alpha 1; HCN2, hyperpolarization-activated cyclic nucleotide-gated ion channel 2; HSP, heat shock protein; IGF-1, insulin-like growth factor-1; IRX5, Iroquois homeobox domain 5; KCND2, Shal-related family member 2; KCNJ2, potassium voltage-gated channel subfamily J member 2; MMP2, matrix metalloproteinase 2; NCX1, sodium–calcium exchanger; PTP1B, protein tyrosine phosphatase 1B; TGF-β, transforming growth factor-β; TRAF5, tumor necrosis factor-related receptor 5; TRPM7, transient receptor potential melastatin 7; TXNIP, thioredoxin-interacting protein. Black arrow indicates promotion, while red arrow indicates inhibition.

These miRNA effects are depicted in summary in Figure 2.

### 1.4. Circulating miRNAs

#### 1.4.1. Stability, Packaging, and Targets of Circulating miRNAs

Extracellular miRNAs in circulation are remarkably stable by being in a vesicle or by forming a complex with proteins. Lipid membrane encapsulation or protein complex renders circulating miRNAs protection against degradation by RNase [74]. Circulating miRNAs can be categorized as vesicle-associated or non-vesicle-associated [75]. Vesicle-associated miRNAs include exosomes (50–90 m) and microvesicles (1 μm) that can be isolated and detected from extracellular fluid [76]. Non-vesicle-associated circulating miRNAs mainly exist as a ribonucleoprotein by making a complex with either Argonaute2, nucleophosmin1, GW182, or high-density lipoprotein (HDL). Due to this complex formation with proteins, this type of miRNAs is protected from RNase unless dissociated from proteins [75].

**Figure 3:**
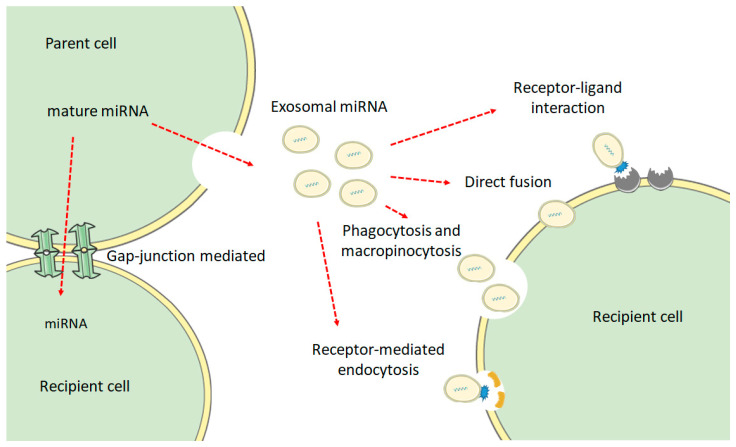
Proposed mechanisms of exosomal microRNA delivery into the target cell.

There has been a growing interest in extracellular vesicles, particularly exosomes in cell communication. Exosomal content comprises of proteins, lipid metabolites, and nucleic acids, including miRNAs [77]. Although argued, several mechanisms explaining the delivery of bioactive cargo of exosomes into a target cell have been proposed [78,79,80]: (a) receptor–ligand interactions, where proteins on exosomal membrane bind with receptors on the target cell membrane, thus stimulating various intracellular signaling cascades in the recipient cell; (b) direct delivery of exosomal cargo into the cytosol by fusion of exosomal membrane with the plasma membrane of target cell; (c) phagocytosis and macropinocytosis; (d) receptor-mediated endocytosis through clathrin- or caveolin-mediated endocytosis; and (e) cell gap junction-mediated transfer between cells. These mechanisms are illustrated in Figure 3. Once inside the target cell, how miRNA and other exosomal bioactive cargoes are protected against degradation by intrinsic endosomal pathways is unclear. Exosomal peptides and lipid metabolites have been known to ultimately end up in the target cell membrane. This indicates the potential fusion of the exosomal cargo with endosomes [78]. However, further research is needed to explain the protection afforded to small RNA material. Exosome research faces several challenges, such as co-isolation of non-exosomal complexes, difficulties in labeling endogenous exosomes to trace their movement and uptake, and issues in identifying recipient cells without impacting cellular function [78].

#### 1.4.2. Circulating miRNAs as a Diagnostic and Prognostic Marker

Due to the robust stability and easy detectability of circulating miRNAs in blood, they are emerging as an attractive biomarker in cardiac injury and remodeling. miR-1, miR-133, miR-208, and miR-499 are intensively being investigated as biomarkers for acute myocardial infarction [42]. Although the accuracy of a single miRNA in detecting myocardial injury is poor, a panel of multiple miRNAs or a combination with cardiac troponin improves the diagnostic power [81]. Circulating miRNAs can help differentiate between heart failure (HF) with preserved and reduced ejection fraction [82]. They can also indicate the progression or reverse of cardiac function. The levels of miR-1, miR-208, and miR-499 normalize after the commencement of the left ventricular assist device in advanced HF [83]. Arrhythmias arising out of electrical abnormalities due to cardiac remodeling are also associated with miRNAs. Compared with healthy subjects, patients with persistent atrial fibrillation have lower plasma miR-150 levels [84]. Despite the promising nature of circulating miRNAs as biomarkers, certain challenges preclude their immediate application in diagnosis or prognosis. The ubiquitous expression makes it difficult to discriminate cardiac miRNAs from muscle and from one clinical condition to another [85]. It is also unclear whether increased levels indicate more expression or increased release from the injured myocardium [86].

#### 1.4.3. Circulating miRNAs and Cardiac Remodeling

Several studies have tried to identify the useful or detrimental effect of exosomal miRNAs on pathological cardiac remodeling after MI. The recently approved heart failure drug sacubitril/valsartan, a neprilysin inhibitor and angiotensin receptor blocker, improves cardiac function and reverses myocardial remodeling [87]. Modulation of miRNA content in plasma exosomes could explain its cardiac effects. Sacubitril/valsartan has been demonstrated to increase the exosomal production in vitro in induced pluripotent stem cell-derived cardiomyocytes and in an in vivo MI model. Next-generation sequencing analysis revealed downregulated miR-181a in these exosomes from the sacubitril/valsartan group [88]. Bone marrow mesenchymal stem cell-derived exosomes were found to shuttle miR-185 to the myocardium to reduce ventricular remodeling and improve cardiac function by reducing the expression of suppressor of cytokine signaling (SOCS2) [89]. miR-21 in exosomes from cardiac stromal cells facilitate heart repair through the phosphatase and tensin homolog/Akt pathway-mediated angiogenesis and cardiomyocyte survival [90]. miRNA array, transcriptomic analysis, and next-generation sequencing have shown miR-19a in exosomal cargo to improve systolic function, induce angiogenesis, and attenuate myocardial fibrosis post-infarction [91]. Conversely, some exosomal miRNAs have also been known to adversely impact cardiac remodeling. miR-142 in exosomes from activated CD4^+^ T cells induces cardiac fibrosis and dysfunction post-infarction through WNT signaling pathway-mediated myofibroblast activation [92]. Cardiac fibroblast-derived exosomal miR-27a, miR-28a, miR-34, miR-130a, and miR-328 dysregulate the Nrf2/ARE signaling cascade and enhance cardiac fibrosis, thus promoting myocardial remodeling [93]. Further research will help in dissecting out targets and developing strategies to either enhance or silence certain exosomal miRNAs to ameliorate cardiac remodeling post-infarction.

### 1.5. Diet, MI, miRNAs, and Future Directions

According to the INTERHEART study, diet is a major modifiable risk factor for MIs, as higher consumption of fruits and vegetables is associated with a reduced risk of an MI [94]. A similar inverse relationship between a healthy diet and a lower risk for cardiovascular disease has been observed in the Lyon Diet Heart Study [95] and the PREDIMED trial [96]. Many nutritional components in heart-healthy diets have been demonstrated to regulate the expression of diverse miRNAs [97]. Ma et al. [98] reported that omega-3 polyunsaturated fatty acids (n-3 PUFAs) protect cardiomyocytes following an MI through the upregulation of anti-apoptotic miRNAs and downregulation of proapoptotic miRNAs. The supplementation of tomato or its rich carotenoid constituent, lycopene, improved diastolic dysfunction, and ameliorated cardiac remodeling through modulation of 8 miRNAs after an MI in rats [99]. Pretreatment with resveratrol, a plant polyphenol, has also been reported to protect against cardiac ischemia/reperfusion injury via modulation of 25 different miRNAs [100]. Selective changes in miRNAs have been reported 8 weeks after an MI in rats on flax oil-supplemented diet rich in n-3 PUFA, alpha-linolenic acid [101]. These changes were associated with cardiac pathological remodeling processes, including apoptosis, fibrosis, and arrhythmias after the MI [102]. Although several miRNAs are now known to control various pathological consequences of an MI, the knowledge regarding the nutritional constituents modulating the expression of various miRNAs following an MI is limited. Therefore, further studies are warranted to examine and dissect molecular miRNA targets of dietary compounds showing therapeutic effect in MIs.

### 1.6. Limitations of Using microRNA as a Therapeutic

Despite being widely studied and shown to be effective in experiments, there are some limitations to the current status of miRNAs as prospective therapeutics. miRNAs and inhibitors of miRNAs do not affect the existing proteins but rather alter the levels of mRNA and their translation. Therefore, it would be necessary to understand the pharmacokinetics of any proposed miRNA therapy [33]. Studies are also required to develop stable anti-miRNAs that can achieve therapeutic efficacy in the heart and to better prevent any off-target effects [33]. Further, the overexpression of any miRNAs for therapeutic purposes requires viral approaches, which would pose a potential challenge for any translational studies in humans [2]. Finally, in some cases, any attempts at genetic deletions have lacked a therapeutic response compared to the short-term inhibition of specific miRNAs [103]. Long-term and carefully designed studies in the future will help understand the reason for the disparate outcomes and aid in the development of efficacious miRNA therapies for cardiovascular pathologies. 

### 1.7. Summary

MicroRNAs are emerging as therapeutic target options for impaired heart rhythms and hemodynamic function. Unlike the standard pharmacological agents that target single molecules in a pathological pathway, miRNAs can interact with several mRNAs and regulate multiple downstream mediators affecting several signaling cascades together [2]. Circulating miRNAs in exosomes can exert action on distant organs and can be exploited for modulating the expression of cardioprotective miRNAs in the heart. In particular, miR -1, -133, -29, and -135 could be investigated further for their beneficial actions on regulating the function of several ion channels, inhibiting cardiac apoptosis, and improving cardiac function.

## Figures and Tables

**Table 1:** microRNAs involved in cardiac remodeling.

MicroRNA	Sample	Model	Sex	Effect on Expression	Target	Reference
miR-101	Sprague–Dawley rats	MI	Male and female	Decreases	RUNX1	[17]
miR-132	miR-132 KO and WT mice	MI	Male	Decreases		[18]
miR-21	miR-21 KO and WT mice	MI	Male	Increases	KBTBD7	[19]
miR-145	miR-145 KO and WT mice	MI	Male	Decreases	KLF4	[20]
miR-26b	Mice	MI	Male	Decreases	PTGS2	[21]
miR-146	CTO patient plasma Mice Pigs	MI	Male and female	Increases	IRAK1CEACAM1	[22]
miR-22	Mice	MI	Male	Increases	Cav3	[23]
miR-330	Mice	MIRI	Male	Increases	SRY	[24]
miR-202	Sprague–Dawley rats	MIRI	Male	Decreases	TRPM6	[25]
miR-30e	Sprague–Dawley rats	MIRI	Male	Decreases	SOX9	[26]
miR-23a	Patient blood	MI	Male	Decreased	PTEN	[27]
miR-143	Patients	MI	Male and female	Increased	SPRY3	[28]
miR-124	Patient plasma Mice	MI		Increased	DHCR2	[29]
miR-19a/b	Patients Mice	MI	Male	Increased	SOCS1	[30]

Cav3, caveolin-3; CEACAM1, carcinoembryonic antigen-related cell adhesion molecule 1; CTO, chronic total occlusion; DHCR2, 3β-hydroxysteroid-delta24 reductase; IRAK1, interleukin 1 receptor associated kinase 1; KBTBD7, kelch repeat and BTB (POZ) domain containing 7; KLF4, Krueppel-like factor 4; KO, knockout; MIRI, myocardial ischemia–reperfusion injury; PTEN, phosphatase and tensin homolog; PTGS2, prostaglandin endoperoxide synthase 2; RUNX1, runt-related transcription factor 1; SOCS1, suppressor of cytokine signaling 1; SOX9, SRY-related high mobility group-box gene 9; SPRY3, sprouty3; SRY, sex-determining region Y; TRPM6, transient receptor potential cation channel, subfamily M, member 6; WT, wildtype.

## References

[1] Liu X., Zhang Y., Du W., Liang H., He H., Zhang L., Pan Z., Li X., Xu C., Zhou Y. (2016). MiR-223–3p as a Novel MicroRNA Regulator of Expression of Voltage-Gated K+ Channel Kv4.2 in Acute Myocardial Infarction. Cell. Physiol. Biochem..

[2] Fiedler J., Thum T. (2013). MicroRNAs in myocardial infarction. Arterioscler. Thromb. Vasc. Biol..

[3] Kim G.H. (2013). MicroRNA regulation of cardiac conduction and arrhythmias. Transl. Res..

[4] Han J., Lee Y., Yeom K.-H., Kim Y.-K., Jin H., Kim V.N. (2004). The Drosha-DGCR8 complex in primary microRNA processing. Genes Dev..

[5] van Rooij E., Olson E.N. (2007). MicroRNAs: Powerful new regulators of heart disease and provocative therapeutic targets. J. Clin. Investig..

[6] Zhou S.S., Jin J.P., Wang J.Q., Zhang Z.G., Freedman J.H., Zheng Y., Cai L. (2018). miRNAS in cardiovascular diseases: Potential biomarkers, therapeutic targets and challenges. Acta Pharmacol. Sin..

[7] Vasudevan S. (2012). Posttranscriptional Upregulation by MicroRNAs. WIREs RNA.

[8] Valinezhad Orang A., Safaralizadeh R., Kazemzadeh-Bavili M. (2014). Mechanisms of miRNA-Mediated Gene Regulation from Common Downregulation to mRNA-Specific Upregulation. Int. J. Genom..

[9] Condorelli G., Latronico M.V.G., Cavarretta E. (2014). microRNAs in Cardiovascular Diseases: Current Knowledge and the Road Ahead. J. Am. Coll. Cardiol..

[10] Creemers E.E., Tijsen A.J., Pinto Y.M., Rooij E.v. (2012). Circulating MicroRNAs. Circ. Res..

[11] Zhu H., Fan G.C. (2012). Role of microRNAs in the reperfused myocardium towards post-infarct remodelling. Circ. Res..

[12] Lionetti V., Tuana B.S., Casieri V., Parikh M., Pierce G.N. (2019). Importance of functional food compounds in cardioprotection through action on the epigenome. Eur. Heart J..

[13] Chistiakov D.A., Orekhov A.N., Bobryshev Y.V. (2016). Cardiac-specific miRNA in cardiogenesis, heart function, and cardiac pathology (with focus on myocardial infarction). J. Mol. Cell. Cardiol..

[14] He B., Xiao J., Ren A.-J., Zhang Y.-F., Zhang H., Chen M., Xie B., Gao X.-G., Wang Y.-W. (2011). Role of miR-1 and miR-133a in myocardial ischemic postconditioning. J. Biomed. Sci..

[15] Dong S., Cheng Y., Yang J., Li J., Liu X., Wang X., Wang D., Krall T.J., Delphin E.S., Zhang C. (2009). MicroRNA expression signature and the role of microRNA-21 in the early phase of acute myocardial infarction. J. Biol. Chem..

[16] Bostjancic E., Zidar N., Glavac D. (2009). MicroRNA microarray expression profiling in human myocardial infarction. Dis. Markers.

[17] Li X., Zhang S., Wa M., Liu Z., Hu S. (2019). MicroRNA-101 Protects Against Cardiac Remodeling Following Myocardial Infarction via Downregulation of Runt-Related Transcription Factor 1. J. Am. Heart Assoc..

[18] Chen L., Wang G.Y., Dong J.H., Cheng X.J. (2019). MicroRNA-132 improves myocardial remodeling after myocardial infarction. Eur. Rev. Med. Pharmacol. Sci..

[19] Yang L., Wang B., Zhou Q., Wang Y., Liu X., Liu Z., Zhan Z. (2018). MicroRNA-21 prevents excessive inflammation and cardiac dysfunction after myocardial infarction through targeting KBTBD7. Cell Death Dis..

[20] Song H.-F., He S., Li S.-H., Wu J., Yin W., Shao Z., Du G.-q., Wu J., Li J., Weisel R.D. (2020). Knock-out of MicroRNA 145 impairs cardiac fibroblast function and wound healing post-myocardial infarction. J. Cell. Physiol..

[21] Ge Z.-W., Zhu X.-L., Wang B.-C., Hu J.-L., Sun J.-J., Wang S., Chen X.-J., Meng S.-P., Liu L., Cheng Z.-Y. (2019). MicroRNA-26b relieves inflammatory response and myocardial remodeling of mice with myocardial infarction by suppression of MAPK pathway through binding to PTGS2. Int. J. Cardiol..

[22] Liao Y., Li H., Cao H., Dong Y., Gao L., Liu Z., Ge J., Zhu H. (2021). Therapeutic silencing miR-146b-5p improves cardiac remodeling in a porcine model of myocardial infarction by modulating the wound reparative phenotype. Protein Cell.

[23] Zhang L., Yin H., Jiao L., Liu T., Gao Y., Shao Y., Zhang Y., Shan H., Zhang Y., Yang B. (2018). Abnormal Downregulation of Caveolin-3 Mediates the Pro-Fibrotic Action of MicroRNA-22 in a Model of Myocardial Infarction. Cell. Physiol. Biochem..

[24] Liu Z.-Y., Pan H.-W., Cao Y., Zheng J., Zhang Y., Tang Y., He J., Hu Y.-J., Wang C.-L., Zou Q.-C. (2019). Downregulated microRNA-330 suppresses left ventricular remodeling via the TGF-β1/Smad3 signaling pathway by targeting SRY in mice with myocardial ischemia–reperfusion injury. J. Cell. Physiol..

[25] Wu H.-Y., Wu J.-L., Ni Z.-L. (2019). Overexpression of microRNA-202-3p protects against myocardial ischemia-reperfusion injury through activation of TGF-β1/Smads signaling pathway by targeting TRPM6. Cell Cycle.

[26] Cheng N., Li L., Wu Y., Wang M., Yang M., Wei S., Wang R. (2021). microRNA-30e up-regulation alleviates myocardial ischemia-reperfusion injury and promotes ventricular remodeling via SOX9 repression. Mol. Immunol..

[27] Li S., Ren J., Sun Q. (2018). The expression of microRNA-23a regulates acute myocardial infarction in patients and in vitro through targeting PTEN. Mol. Med. Rep..

[28] Li C., Li J., Xue K., Zhang J., Wang C., Zhang Q., Chen X., Gao C., Yu X., Sun L. (2019). MicroRNA-143-3p promotes human cardiac fibrosis via targeting sprouty3 after myocardial infarction. J. Mol. Cell. Cardiol..

[29] Han F., Chen Q., Su J., Zheng A., Chen K., Sun S., Wu H., Jiang L., Xu X., Yang M. (2019). MicroRNA-124 regulates cardiomyocyte apoptosis and myocardial infarction through targeting Dhcr24. J. Mol. Cell. Cardiol..

[30] Gao F., Kataoka M., Liu N., Liang T., Huang Z.-P., Gu F., Ding J., Liu J., Zhang F., Ma Q. (2019). Therapeutic role of miR-19a/19b in cardiac regeneration and protection from myocardial infarction. Nat. Commun..

[31] Liao C., Gui Y., Guo Y., Xu D. (2016). The regulatory function of microRNA-1 in arrhythmias. Mol. Biosyst..

[32] Yang B., Lin H., Xiao J., Lu Y., Luo X., Li B., Zhang Y., Xu C., Bai Y., Wang H. (2007). The muscle-specific microRNA miR-1 regulates cardiac arrhythmogenic potential by targeting GJA1 and KCNJ2. Nat. Med..

[33] Mitra M., Coller H.A. (2016). RNAs that make a heart beat. Ann. Transl. Med..

[34] Lu Y., Zhang Y., Shan H., Pan Z., Li X., Li B., Xu C., Zhang B., Zhang F., Dong D. (2009). MicroRNA-1 downregulation by propranolol in a rat model of myocardial infarction: A new mechanism for ischaemic cardioprotection. Cardiovasc. Res..

[35] Shan Z.X., Lin Q.X., Fu Y.H., Deng C.Y., Zhou Z.L., Zhu J.N., Liu X.Y., Zhang Y.Y., Li Y., Lin S.G. (2009). Upregulated expression of miR-1/miR-206 in a rat model of myocardial infarction. Biochem. Biophys. Res. Commun..

[36] Li N., Zhou H., Tang Q. (2018). miR-133: A Suppressor of Cardiac Remodeling?. Front. Pharmacol..

[37] Liu Y., Liang Y., Zhang J.F., Fu W.M. (2017). MicroRNA-133 mediates cardiac diseases: Mechanisms and clinical implications. Exp. Cell Res..

[38] Bostjancic E., Zidar N., Stajner D., Glavac D. (2010). MicroRNA miR-1 is up-regulated in remote myocardium in patients with myocardial infarction. Folia. Biol. (Praha).

[39] Li S., Xiao F.Y., Shan P.R., Su L., Chen D.L., Ding J.Y., Wang Z.Q. (2015). Overexpression of microRNA-133a inhibits ischemia-reperfusion-induced cardiomyocyte apoptosis by targeting DAPK2. J. Hum. Genet..

[40] Xu C., Hu Y., Hou L., Ju J., Li X., Du N., Guan X., Liu Z., Zhang T., Qin W. (2014). beta-Blocker carvedilol protects cardiomyocytes against oxidative stress-induced apoptosis by up-regulating miR-133 expression. J. Mol. Cell. Cardiol..

[41] Bostjancic E., Zidar N., Stajer D., Glavac D. (2010). MicroRNAs miR-1, miR-133a, miR-133b and miR-208 are dysregulated in human myocardial infarction. Cardiology.

[42] D’Alessandra Y., Devanna P., Limana F., Straino S., Di Carlo A., Brambilla P.G., Rubino M., Carena M.C., Spazzafumo L., De Simone M. (2010). Circulating microRNAs are new and sensitive biomarkers of myocardial infarction. Eur. Heart J..

[43] Kuwabara Y., Ono K., Horie T., Nishi H., Nagao K., Kinoshita M., Watanabe S., Baba O., Kojima Y., Shizuta S. (2011). Increased microRNA-1 and microRNA-133a levels in serum of patients with cardiovascular disease indicate myocardial damage. Circ. Cardiovasc. Genet..

[44] Wang G.K., Zhu J.Q., Zhang J.T., Li Q., Li Y., He J., Qin Y.W., Jing Q. (2010). Circulating microRNA: A novel potential biomarker for early diagnosis of acute myocardial infarction in humans. Eur. Heart J..

[45] Chen Y., Zhao Y., Chen W., Xie L., Zhao Z.-A., Yang J., Chen Y., Lei W., Shen Z. (2017). MicroRNA-133 overexpression promotes the therapeutic efficacy of mesenchymal stem cells on acute myocardial infarction. Stem Cell Res. Ther..

[46] Chen S., Puthanveetil P., Feng B., Matkovich S.J., Dorn G.W., Chakrabarti S. (2014). Cardiac miR-133a overexpression prevents early cardiac fibrosis in diabetes. J. Cell. Mol. Med..

[47] Sang H.-Q., Jiang Z.-M., Zhao Q.-P., Xin F. (2015). MicroRNA-133a improves the cardiac function and fibrosis through inhibiting Akt in heart failure rats. Biomed. Pharmacother..

[48] Castoldi G., Di Gioia C.R., Bombardi C., Catalucci D., Corradi B., Gualazzi M.G., Leopizzi M., Mancini M., Zerbini G., Condorelli G. (2012). MiR-133a regulates collagen 1A1: Potential role of miR-133a in myocardial fibrosis in angiotensin II-dependent hypertension. J. Cell. Physiol..

[49] Ong S.-B., Hernández-Reséndiz S., Crespo-Avilan G.E., Mukhametshina R.T., Kwek X.-Y., Cabrera-Fuentes H.A., Hausenloy D.J. (2018). Inflammation following acute myocardial infarction: Multiple players, dynamic roles, and novel therapeutic opportunities. Pharmacol. Ther..

[50] Xiao Y., Zhao J., Tuazon J.P., Borlongan C.V., Yu G. (2019). MicroRNA-133a and Myocardial Infarction. Cell Transplant..

[51] Muraoka N., Yamakawa H., Miyamoto K., Sadahiro T., Umei T., Isomi M., Nakashima H., Akiyama M., Wada R., Inagawa K. (2014). MiR-133 promotes cardiac reprogramming by directly repressing Snai1 and silencing fibroblast signatures. EMBO Rep..

[52] Bostjancic E., Brandner T., Zidar N., Glavac D., Stajer D. (2018). Down-regulation of miR-133a/b in patients with myocardial infarction correlates with the presence of ventricular fibrillation. Biomed. Pharmacother..

[53] van Rooij E., Sutherland L.B., Thatcher J.E., DiMaio J.M., Naseem R.H., Marshall W.S., Hill J.A., Olson E.N. (2008). Dysregulation of microRNAs after myocardial infarction reveals a role of miR-29 in cardiac fibrosis. Proc. Natl. Acad. Sci. USA..

[54] Zhao W., Cheng L., Quek C., Bellingham S.A., Hill A.F. (2019). Novel miR-29b target regulation patterns are revealed in two different cell lines. Sci. Rep..

[55] Jing X., Yang J., Jiang L., Chen J., Wang H. (2018). MicroRNA-29b Regulates the Mitochondria-Dependent Apoptotic Pathway by Targeting Bax in Doxorubicin Cardiotoxicity. Cell. Physiol. Biochem..

[56] Wang Y., Jin B.J., Chen Q., Yan B.J., Liu Z.L. (2019). MicroRNA-29b upregulation improves myocardial fibrosis and cardiac function in myocardial infarction rats through targeting SH2B3. Eur. Rev. Med. Pharmacol. Sci..

[57] Kriegel A.J., Liu Y., Fang Y., Ding X., Liang M. (2012). The miR-29 family: Genomics, cell biology, and relevance to renal and cardiovascular injury. Physiol. Genom..

[58] Zhang Y., Huang X.R., Wei L.H., Chung A.C., Yu C.M., Lan H.Y. (2014). miR-29b as a therapeutic agent for angiotensin II-induced cardiac fibrosis by targeting TGF-beta/Smad3 signaling. Mol. Ther..

[59] Zhu J.-N., Chen R., Fu Y.-H., Lin Q.-X., Huang S., Guo L.-L., Zhang M.-Z., Deng C.-Y., Zou X., Zhong S.-L. (2013). Smad3 Inactivation and MiR-29b Upregulation Mediate the Effect of Carvedilol on Attenuating the Acute Myocardium Infarction-Induced Myocardial Fibrosis in Rat. PLoS ONE.

[60] Sassi Y., Avramopoulos P., Ramanujam D., Gruter L., Werfel S., Giosele S., Brunner A.D., Esfandyari D., Papadopoulou A.S., De Strooper B. (2017). Cardiac myocyte miR-29 promotes pathological remodeling of the heart by activating Wnt signaling. Nat. Commun..

[61] Zhu M.L., Yin Y.L., Ping S., Yu H.Y., Wan G.R., Jian X., Li P. (2017). Berberine promotes ischemia-induced angiogenesis in mice heart via upregulation of microRNA-29b. Clin. Exp. Hypertens..

[62] Travers J.G., Kamal F.A., Robbins J., Yutzey K.E., Blaxall B.C. (2016). Cardiac Fibrosis: The Fibroblast Awakens. Circ. Res..

[63] Cai Y., Li Y. (2019). Upregulation of miR-29b-3p protects cardiomyocytes from hypoxia-induced apoptosis by targeting TRAF5. Cell Mol. Biol. Lett..

[64] van der Laan A.M., Piek J.J., van Royen N. (2009). Targeting angiogenesis to restore the microcirculation after reperfused MI. Nat. Rev. Cardiol..

[65] Issler O., Haramati S., Paul E.D., Maeno H., Navon I., Zwang R., Gil S., Mayberg H.S., Dunlop B.W., Menke A. (2014). MicroRNA 135 Is Essential for Chronic Stress Resiliency, Antidepressant Efficacy, and Intact Serotonergic Activity. Neuron.

[66] Nagel R., le Sage C., Diosdado B., van der Waal M., Oude Vrielink J.A.F., Bolijn A., Meijer G.A., Agami R. (2008). Regulation of the Adenomatous Polyposis Coli Gene by the miR-135 Family in Colorectal Cancer. Cancer Res..

[67] Chu Q., Li A., Chen X., Qin Y., Sun X., Li Y., Yue E., Wang C., Ding X., Yan Y. (2018). Overexpression of miR-135b attenuates pathological cardiac hypertrophy by targeting CACNA1C. Int. J. Cardiol..

[68] Li A., Yu Y., Ding X., Qin Y., Jiang Y., Wang X., Liu G., Chen X., Yue E., Sun X. (2019). MiR-135b protects cardiomyocytes from infarction through restraining the NLRP3/caspase-1/IL-1β pathway. Int. J. Cardiol..

[69] Zhu H.-J., Wang D.-G., Yan J., Xu J. (2015). Up-regulation of microRNA-135a protects against myocardial ischemia/reperfusion injury by decreasing TXNIP expression in diabetic mice. Am. J. Transl. Res..

[70] Wang B.F., Yoshioka J. (2017). The Emerging Role of Thioredoxin-Interacting Protein in Myocardial Ischemia/Reperfusion Injury. J. Cardiovasc. Pharmacol. Ther..

[71] Wang S., Cheng Z., Chen X., Xue H. (2019). microRNA-135a protects against myocardial ischemia-reperfusion injury in rats by targeting protein tyrosine phosphatase 1B. J. Cell. Biochem..

[72] Wu Y., Liu Y., Pan Y., Lu C., Xu H., Wang X., Liu T., Feng K., Tang Y. (2018). MicroRNA-135a inhibits cardiac fibrosis induced by isoproterenol via TRPM7 channel. Biomed. Pharmacother..

[73] Duong E., Xiao J., Qi X.Y., Nattel S. (2017). MicroRNA-135a regulates sodium-calcium exchanger gene expression and cardiac electrical activity. Heart Rhythm.

[74] Mitchell P.S., Parkin R.K., Kroh E.M., Fritz B.R., Wyman S.K., Pogosova-Agadjanyan E.L., Peterson A., Noteboom J., O’Briant K.C., Allen A. (2008). Circulating microRNAs as stable blood-based markers for cancer detection. Proc. Natl. Acad. Sci. USA.

[75] Cui M., Wang H., Yao X., Zhang D., Xie Y., Cui R., Zhang X. (2019). Circulating MicroRNAs in Cancer: Potential and Challenge. Front. Genet..

[76] Hunter M.P., Ismail N., Zhang X., Aguda B.D., Lee E.J., Yu L., Xiao T., Schafer J., Lee M.L., Schmittgen T.D. (2008). Detection of microRNA expression in human peripheral blood microvesicles. PloS ONE.

[77] Valadi H., Ekström K., Bossios A., Sjöstrand M., Lee J.J., Lötvall J.O. (2007). Exosome-mediated transfer of mRNAs and microRNAs is a novel mechanism of genetic exchange between cells. Nat. Cell Biol..

[78] Huang-Doran I., Zhang C.-Y., Vidal-Puig A. (2017). Extracellular Vesicles: Novel Mediators of Cell Communication In Metabolic Disease. Trends Endocrinol. Metab..

[79] Lim P.K., Bliss S.A., Patel S.A., Taborga M., Dave M.A., Gregory L.A., Greco S.J., Bryan M., Patel P.S., Rameshwar P. (2011). Gap junction-mediated import of microRNA from bone marrow stromal cells can elicit cell cycle quiescence in breast cancer cells. Cancer Res..

[80] Aucher A., Rudnicka D., Davis D.M. (2013). MicroRNAs transfer from human macrophages to hepato-carcinoma cells and inhibit proliferation. J. Immunol..

[81] Zeller T., Keller T., Ojeda F., Reichlin T., Twerenbold R., Tzikas S., Wild P.S., Reiter M., Czyz E., Lackner K.J. (2014). Assessment of microRNAs in patients with unstable angina pectoris. Eur. Heart J..

[82] Watson C.J., Gupta S.K., O’Connell E., Thum S., Glezeva N., Fendrich J., Gallagher J., Ledwidge M., Grote-Levi L., McDonald K. (2015). MicroRNA signatures differentiate preserved from reduced ejection fraction heart failure. Eur. J. Heart Fail..

[83] Akat K.M., Moore-McGriff D., Morozov P., Brown M., Gogakos T., Correa Da Rosa J., Mihailovic A., Sauer M., Ji R., Ramarathnam A. (2014). Comparative RNA-sequencing analysis of myocardial and circulating small RNAs in human heart failure and their utility as biomarkers. Proc. Natl. Acad. Sci. USA.

[84] McManus D.D., Tanriverdi K., Lin H., Esa N., Kinno M., Mandapati D., Tam S., Okike O.N., Ellinor P.T., Keaney J.F. (2015). Plasma microRNAs are associated with atrial fibrillation and change after catheter ablation (the miRhythm study). Heart Rhythm.

[85] Small E.M., Olson E.N. (2011). Pervasive roles of microRNAs in cardiovascular biology. Nature.

[86] Min P.K., Chan S.Y. (2015). The biology of circulating microRNAs in cardiovascular disease. J. Clin. Investig..

[87] Januzzi J.L., Camacho A., Piña I.L., Rocha R., Williamson K.M., Maisel A.S., Felker G.M., Prescott M.F., Butler J., Solomon S.D. (2020). Reverse Cardiac Remodeling and Outcome After Initiation of Sacubitril/Valsartan. Circ. Heart Fail..

[88] Vaskova E., Ikeda G., Tada Y., Wahlquist C., Mercola M., Yang P.C. (2020). Sacubitril/Valsartan Improves Cardiac Function and Decreases Myocardial Fibrosis Via Downregulation of Exosomal miR-181a in a Rodent Chronic Myocardial Infarction Model. J. Am. Heart Assoc..

[89] Li Y., Zhou J., Zhang O., Wu X., Guan X., Xue Y., Li S., Zhuang X., Zhou B., Miao G. (2020). Bone marrow mesenchymal stem cells-derived exosomal microRNA-185 represses ventricular remolding of mice with myocardial infarction by inhibiting SOCS2. Int. Immunopharmacol..

[90] Qiao L., Hu S., Liu S., Zhang H., Ma H., Huang K., Li Z., Su T., Vandergriff A., Tang J. (2019). microRNA-21-5p dysregulation in exosomes derived from heart failure patients impairs regenerative potential. J. Clin. Investig..

[91] Gollmann-Tepeköylü C., Pölzl L., Graber M., Hirsch J., Nägele F., Lobenwein D., Hess M.W., Blumer M.J., Kirchmair E., Zipperle J. (2020). miR-19a-3p containing exosomes improve function of ischaemic myocardium upon shock wave therapy. Cardiovasc. Res..

[92] Cai L., Chao G., Li W., Zhu J., Li F., Qi B., Wei Y., Chen S., Zhou G., Lu X. (2020). Activated CD4(+) T cells-derived exosomal miR-142-3p boosts post-ischemic ventricular remodeling by activating myofibroblast. Aging.

[93] Tikhomirov R., Donnell B.R., Catapano F., Faggian G., Gorelik J., Martelli F., Emanueli C. (2020). Exosomes: From Potential Culprits to New Therapeutic Promise in the Setting of Cardiac Fibrosis. Cells.

[94] Iqbal R., Anand S., Ounpuu S., Islam S., Zhang X., Rangarajan S., Chifamba J., Al-Hinai A., Keltai M., Yusuf S. (2008). Dietary Patterns and the Risk of Acute Myocardial Infarction in 52 Countries. Circulation.

[95] de Lorgeril M., Salen P., Martin J.L., Monjaud I., Delaye J., Mamelle N. (1999). Mediterranean diet, traditional risk factors, and the rate of cardiovascular complications after myocardial infarction: Final report of the Lyon Diet Heart Study. Circulation.

[96] Estruch R., Ros E., Salas-Salvadó J., Covas M.I., Corella D., Arós F., Gómez-Gracia E., Ruiz-Gutiérrez V., Fiol M., Lapetra J. (2018). Primary Prevention of Cardiovascular Disease with a Mediterranean Diet Supplemented with Extra-Virgin Olive Oil or Nuts. N. Engl. J. Med..

[97] Kura B., Parikh M., Slezak J., Pierce G.N. (2019). The Influence of Diet on MicroRNAs that Impact Cardiovascular Disease. Molecules.

[98] Ma H., Chen P., Sang C., Huang D., Geng Q., Wang L. (2018). Modulation of apoptosis-related microRNAs following myocardial infarction in fat-1 transgenic mice vs wild-type mice. J. Cell. Mol. Med..

[99] Pereira B.L.B., Reis P.P., Severino F.E., Felix T.F., Braz M.G., Nogueira F.R., Silva R.A.C., Cardoso A.C., Lourenço M.A.M., Figueiredo A.M. (2017). Tomato (Lycopersicon esculentum) or lycopene supplementation attenuates ventricular remodeling after myocardial infarction through different mechanistic pathways. J. Nutr. Biochem..

[100] Mukhopadhyay P., Mukherjee S., Ahsan K., Bagchi A., Pacher P., Das D.K. (2010). Restoration of altered microRNA expression in the ischemic heart with resveratrol. PLoS ONE.

[101] Parikh M., Kura B., O’Hara K.A., Dibrov E., Netticadan T., Slezak J., Pierce G.N. (2020). Cardioprotective Effects of Dietary Flaxseed Post-Infarction are Associated with Changes in MicroRNA Expression. Biomolecules.

[102] Parikh M., Raj P., Austria J.A., Yu L., Garg B., Netticadan T., Pierce G.N. (2019). Dietary flaxseed protects against ventricular arrhythmias and left ventricular dilation after a myocardial infarction. J. Nutr. Biochem..

[103] Mendell J.T., Olson E.N. (2012). MicroRNAs in stress signaling and human disease. Cell.

